# Delayed Reconstruction of Post-traumatic Enophthalmos and Orbitomaxillary Deformity 25 Years After a Childhood Dog-Bite Injury: A Case Report

**DOI:** 10.7759/cureus.116840

**Published:** 2026-09-24

**Authors:** Ali Altan Ertan Boz

**Affiliations:** 1 Ophthalmology, Yenikent State Hospital, Sakarya, TUR

**Keywords:** dog bite, eyelid reconstruction, maxillary reconstruction, orbital floor reconstruction, orbitomaxillary deformity, post-traumatic enophthalmos

## Abstract

Dog-bite injuries in children usually involve facial soft tissues, while associated craniofacial fractures are uncommon and may be overlooked during the acute phase. A 35-year-old woman presented with long-standing right periocular asymmetry, infraorbital hollowing, anterior maxillary depression, and a medial lower eyelid notch 25 years after a childhood dog-bite injury. Best-corrected visual acuity was 20/20 in both eyes, ocular motility was normal, and diplopia was absent. Hertel exophthalmometry measured 18 mm on the right and 22 mm on the left. Computed tomography demonstrated chronic deformity of the right inferior orbital wall and anterior maxilla. Through a transconjunctival lower orbital approach extended to the anterior maxillary wall, the deformed anterior maxilla was divided vertically into three segments, reconstructed with block allograft bone, and stabilized with titanium plates and screws. The orbital floor was reconstructed with contoured titanium mesh, and the lower eyelid notch was treated by wedge resection and primary repair. At one month, periocular symmetry and midfacial projection had improved; Hertel measurements were 22 mm bilaterally, and postoperative imaging confirmed the position of the orbital mesh and maxillary fixation. This case illustrates the need to evaluate orbital support, midfacial projection, and eyelid contour together when treating chronic combined orbitomaxillary deformity after childhood facial trauma.

## Introduction

Dog-bite injuries are a common cause of pediatric facial trauma because children are more likely than adults to sustain bites to the head and neck. Most facial dog bites involve soft tissues, but fractures of the orbit, maxilla, zygoma, mandible, and skull have been reported [1-6]. In a large pediatric series, facial fractures accompanied 17 of 1,201 facial dog-bite injuries, illustrating that skeletal injury is uncommon but clinically important [3].

The acute literature focuses primarily on wound management, infection prevention, fracture recognition, and early reconstruction. Long-standing skeletal and eyelid sequelae after childhood canine trauma are less often described. When a deformity persists into adulthood, remodeling, scarred soft-tissue planes, orbital volume change, and loss of midfacial projection may need to be addressed simultaneously rather than as an isolated orbital floor problem.

This case report describes delayed reconstruction of anterior maxillary depression with an adjoining orbital floor contour deformity, relative globe recession, and a medial lower eyelid notch in an adult who reported a childhood dog-bite injury 25 years earlier. The educational value of this case is the integrated management of anatomically linked orbital, maxillary, and eyelid deformities during secondary reconstruction.

## Case presentation

A 35-year-old woman presented with long-standing asymmetry of the right periocular and midfacial region. She reported a dog bite to the right side of the face at 10 years of age. Records from the acute childhood injury were unavailable; therefore, the original fracture pattern, initial wound care, antimicrobial treatment, and tetanus or rabies prophylaxis could not be confirmed.

Her principal concern was the persistent contour asymmetry. Examination showed right infraorbital hollowing, anterior maxillary depression, periocular asymmetry, and a medial lower eyelid notch. There was no open wound or active infection. Preoperative best-corrected visual acuity was 20/20 in both eyes. Pupillary reactions and ocular motility were normal, and diplopia was absent. Hertel exophthalmometry measured 18 mm on the right and 22 mm on the left. Anterior and posterior segment examinations were unremarkable.

Preoperative maxillofacial computed tomography demonstrated localized depression, angulation, and step-like contour irregularity at the junction of the right anterior and anterolateral maxillary walls. The adjoining right orbital floor had an irregular, depressed contour. On anatomical assessment, the chronic deformity extended approximately 15 mm posteriorly along the orbital floor. No radiologic evidence of extraocular muscle entrapment was identified (Figure 1).

**Figure 1:**
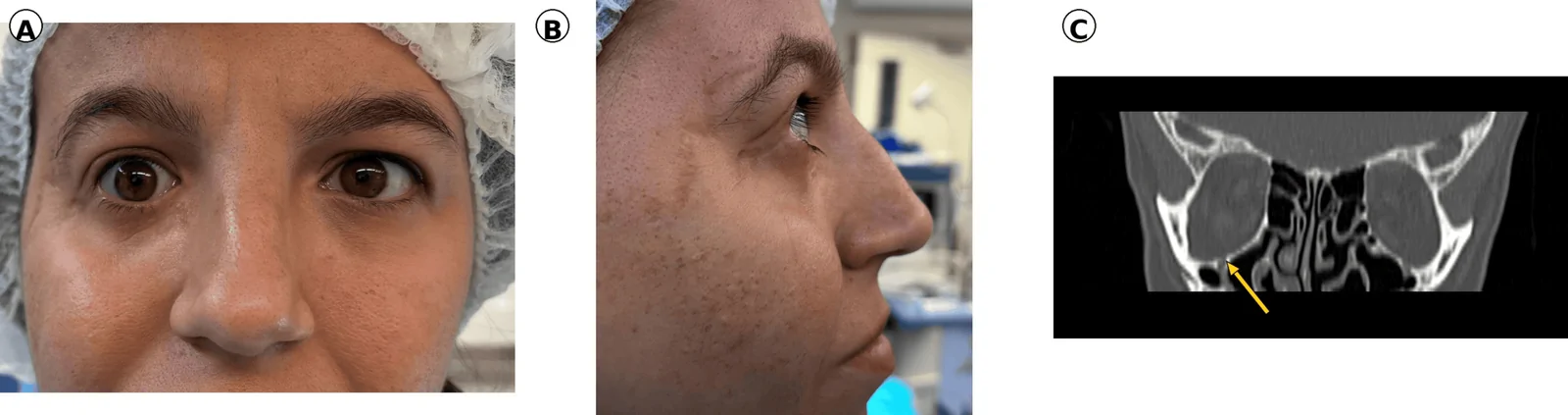
Preoperative Clinical and Computed Tomography Findings. (A) Frontal photograph showing right periocular asymmetry, infraorbital hollowing, and midfacial contour depression. (B) Right profile photograph showing reduced anterior maxillary projection. (C) Coronal computed tomography image showing the depressed, irregular right orbital floor contour adjoining the anterior maxillary deformity (yellow straight arrow). Written informed consent was obtained from the patient for publication of this case report and accompanying images.

Surgery was performed under general anesthesia. A transconjunctival lower orbital approach was used, and the dissection was extended inferiorly to the anterior maxillary wall. No extraocular muscle entrapment was identified intraoperatively. The principal reconstructive objective was correction of the chronic anterior maxillary depression. The deformed anterior maxilla was exposed and divided vertically into three segments to permit controlled mobilization and contour restoration. The intervening gaps were reconstructed with block allograft bone, and the mobilized maxillary segments were stabilized with titanium plates and screws. Contoured titanium mesh was placed to maintain orbital floor support after osteotomy and maxillary segment mobilization. Implant position was checked intraoperatively with attention to avoiding soft-tissue entrapment (Figure 2).

**Figure 2:**
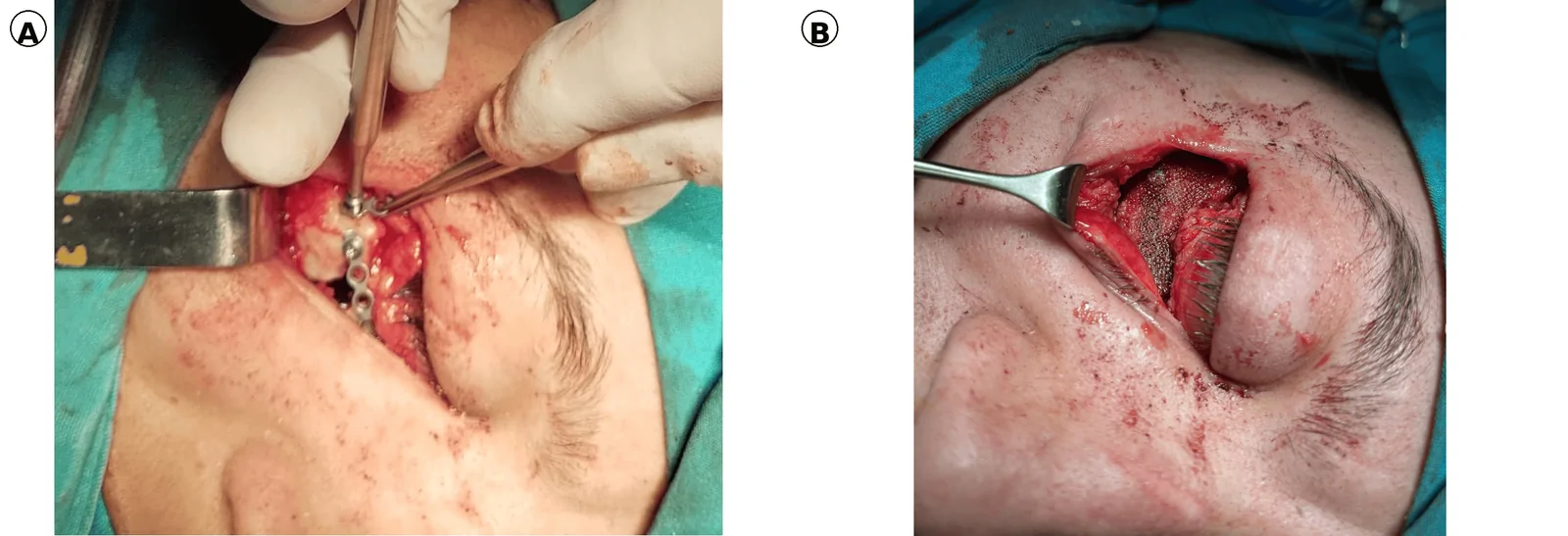
Intraoperative Orbitomaxillary Reconstruction. (A) Block allograft-supported anterior maxillary reconstruction with titanium plate fixation after vertical segmentation of the maxillary wall. (B) Contoured titanium mesh positioned through the lower orbital exposure to maintain right orbital floor support after maxillary segment mobilization.

The medial lower eyelid notch was addressed during the same operation. The irregular eyelid margin was excised by wedge resection and repaired primarily. No intraoperative complication occurred.

Early follow-up showed expected periocular edema and ecchymosis. Serial clinical photographs demonstrated progressive reduction in swelling and improvement in contour. At one month, periocular symmetry and midfacial projection were improved. Hertel exophthalmometry measured 22 mm in each eye, corresponding to a reduction in the interocular difference from 4 mm preoperatively to 0 mm. Extraocular movements remained full in all directions without restriction, and the patient reported no diplopia. The right lower eyelid was in a normal position, with no ectropion or entropion, and the repaired eyelid margin had a regular contour. Eyelid closure was complete without lagophthalmos, and the patient reported no epiphora. Postoperative computed tomography showed the titanium mesh along the reconstructed orbital floor and titanium plate fixation of the anterior maxillary segments (Figure 3).

**Figure 3:**
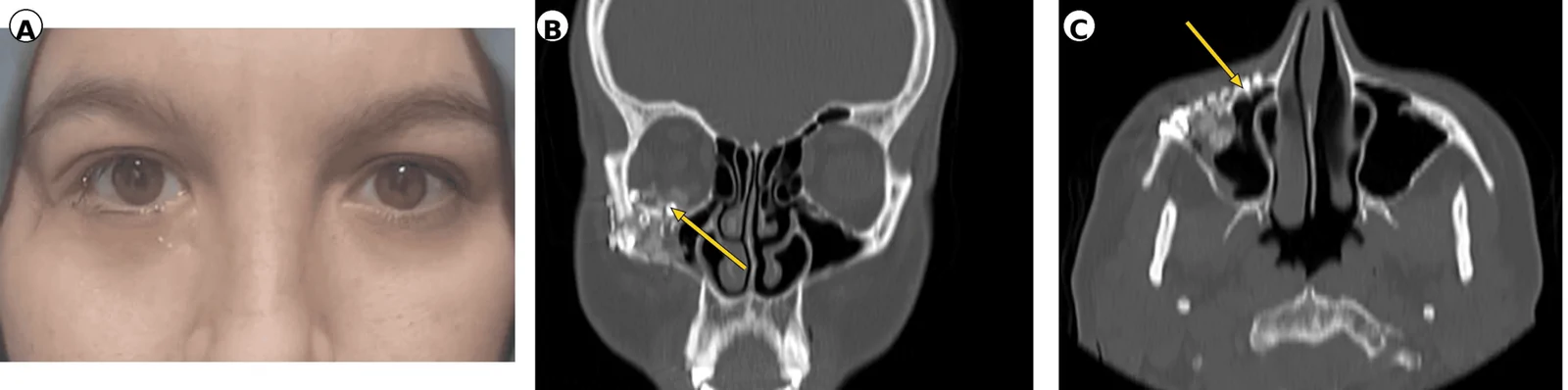
Early Postoperative Clinical and Computed Tomography Findings. (A) Clinical photograph at one month showing improved periocular symmetry and midfacial contour. (B) Coronal computed tomography image showing the titanium mesh supporting the right orbital floor after maxillary reconstruction (yellow straight arrow). (C) Axial computed tomography image showing titanium plate fixation of the reconstructed right anterior maxillary wall (yellow straight arrow). Written informed consent was obtained from the patient for publication of this case report and accompanying images.

Quantitative CT assessment

The preoperative and postoperative computed tomography were assessed quantitatively. A fitted midline and mirrored contralateral cortical reference were used for the preoperative linear measurements. At the selected axial location, the right anterior maxillary cortex showed a local anteroposterior offset of approximately 5.3 mm relative to this reference. On matched parasagittal reformations 25 mm lateral to the midline, the selected right orbital floor point lay approximately 1.9 mm inferior to its contralateral reference. Figure 4 shows these local measurements and postoperative reconstruction views.

**Figure 4:**
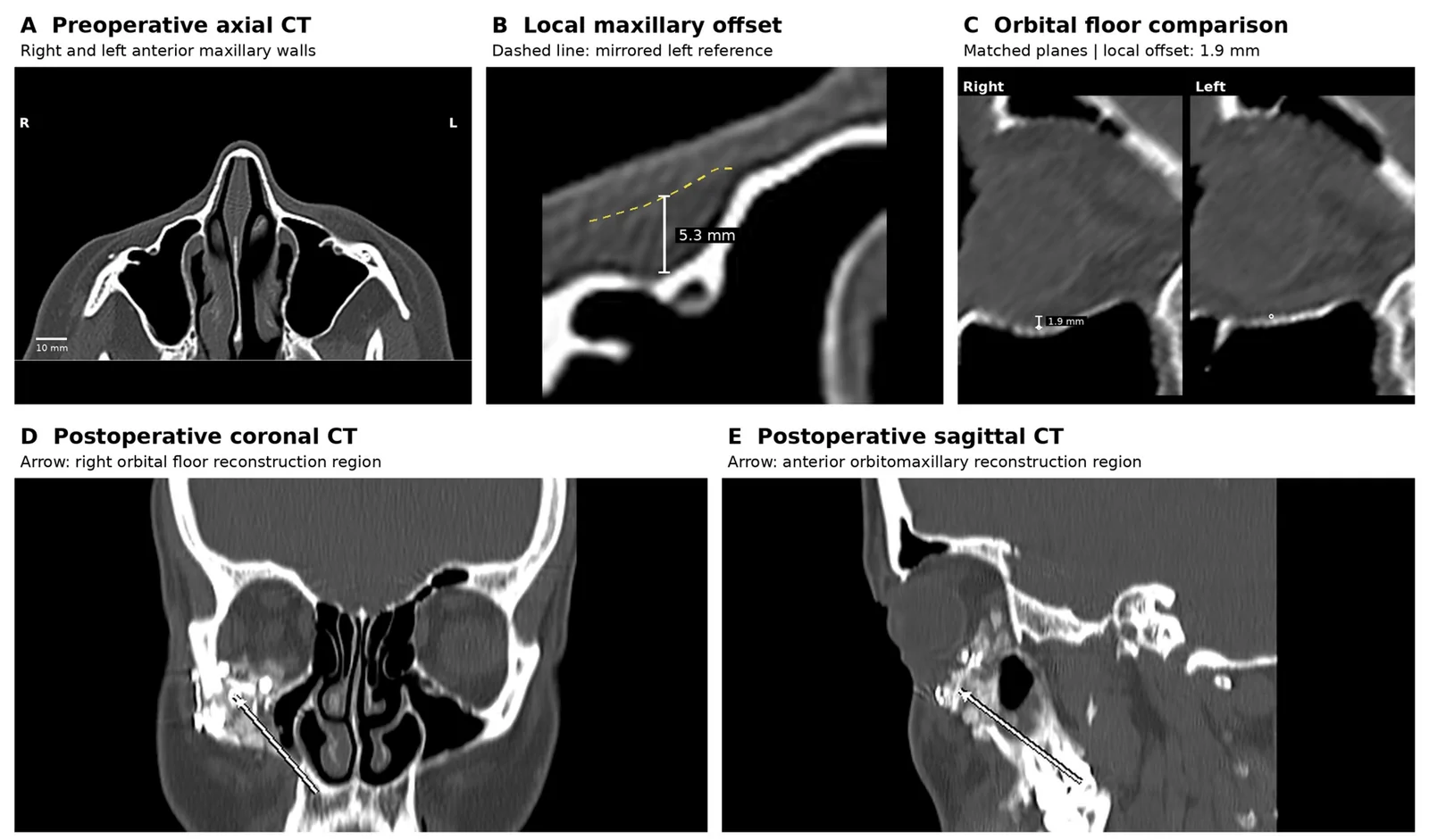
Preoperative and Postoperative Measurements on Computed Tomography. (A) Preoperative axial view of the anterior and anterolateral maxillary walls. (B) Enlarged right maxillary region; the yellow dashed line represents the mirrored contralateral cortical reference, and the white line marks the approximately 5.3-mm local anteroposterior offset. (C) The right orbital floor point is approximately 1.9 mm inferior to its contralateral reference. (D) Postoperative coronal image; the white arrow identifies the right orbital floor reconstruction region. (E) Postoperative sagittal image; the white arrow identifies the anterior orbitomaxillary reconstruction region.

Bilateral orbital compartment volumes were measured with the same segmentation protocol. Medial and lateral orbital rim points defined the anterior closure; posterior stops were placed near the optic canal entrance, and fissures were bounded by the contour envelope. Volume in mL was calculated. The right and left orbital compartment volumes were approximately 17.1 and 16.3 mL preoperatively and 16.1 and 16.3 mL postoperatively, respectively. The right-minus-left difference was approximately +0.76 mL before reconstruction and -0.2 mL afterward. The right compartment decreased by approximately 1.0 mL (5.9%) (Table 1). Matched orthogonal CT sections with the segmentation are shown in Figure 5.

**Table 1:** CT-Segmented Orbital Compartment Volumes and Changes.

Measurement	Preoperative	Postoperative	Change (mL/%)
Right compartment (mL)	17.1	16.1	-1.0/5.9
Left compartment (mL)	16.3	16.3	0.00
Right minus left (mL)	0.76	-0.2	-

**Figure 5:**
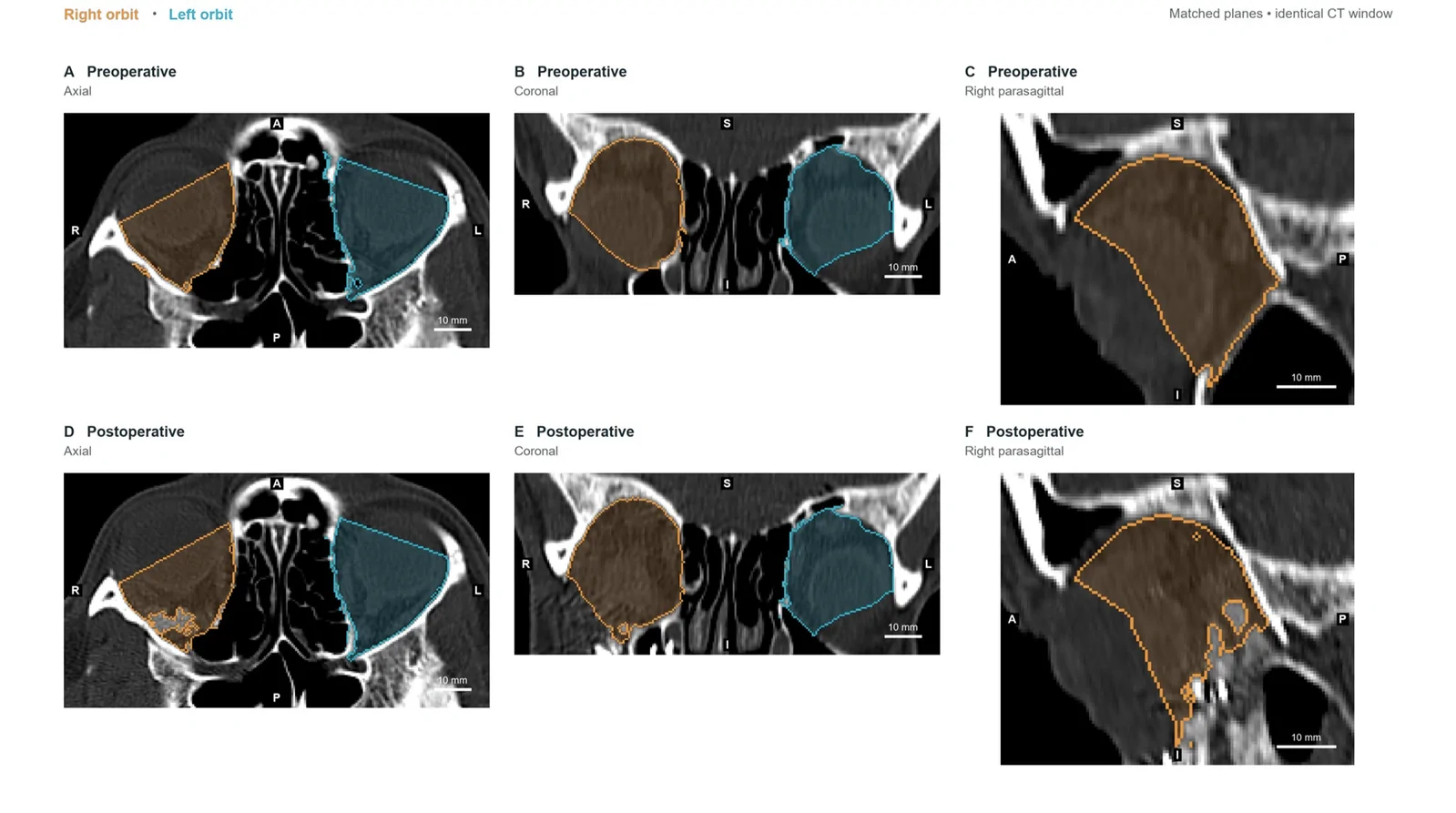
Matched Preoperative and Postoperative CT-Based Orbital Segmentation. Preoperative (A-C) and postoperative (D-F) images show matched axial (A, D), coronal (B, E), and right parasagittal (C, F) sections. Both studies use the same CT window. Orange and cyan contours and translucent overlays identify the right and left measured orbital compartments, respectively. Boundaries follow the orbital-wall contours, rim-based anterior closure, and posterior stops near the optic canal entrance. Tissue anterior to the closure and thresholded air, bone, and metal are excluded.

## Discussion

Facial fractures associated with dog bites are uncommon, but they occur disproportionately in children and can involve the orbit and midface [3-8]. Pediatric facial injuries may initially appear to be dominated by lacerations and soft-tissue loss, while underlying skeletal injury is less obvious. Published reports include maxillary, mandibular, zygomatic, orbital, and panfacial fractures caused by canine attacks [8-12]. The present case differs from most of that literature because the patient presented as an adult with a chronic combined deformity rather than an acute fracture requiring primary repair.

Secondary reconstruction of a long-standing orbitomaxillary deformity differs from acute fracture treatment. The surgeon is not simply returning mobile fracture segments to their original position; the problem may include remodeled bone, altered soft-tissue relationships, and persistent deficiency of midfacial projection. In this patient, the anterior maxillary depression was accompanied by chronic fracture-related irregularity extending into the adjoining orbital floor region and a locally depressed floor contour. Orbital floor support alone would not have corrected the anterior maxillary contour. Conversely, maxillary osteotomy and segment mobilization could affect the adjacent supporting surface beneath the orbital contents. Mesh placement was intended to maintain this support after repositioning rather than to release entrapped muscle. The lower eyelid notch was repaired in the same setting.

Titanium mesh is an established option for orbital floor reconstruction because it provides rigid support and can be contoured to the defect [13-15]. Bone grafting with plate fixation and direct eyelid margin repair are likewise established reconstructive methods. The individual techniques used in this case are therefore not presented as novel procedures. In this patient, these established techniques were combined according to the local anatomy and the support requirements created by maxillary segment mobilization.

The quantitative CT assessment documented local anterior maxillary depression and inferior orbital floor displacement together with a preoperative right orbital compartment volume approximately 0.76 mL greater than the left. After reconstruction, the right compartment volume decreased by approximately 1.0 mL and was approximately 0.2 mL smaller than the left. These findings document the measured orbital compartment volume change associated with reconstruction. The equal postoperative Hertel measurements document clinical globe-position symmetry as a separate outcome. Full ocular motility, absence of diplopia, normal lower eyelid position and contour, complete closure, and absence of epiphora further characterize the early clinical result.

The childhood records and imaging were unavailable, so the original fracture pattern and details of acute skeletal treatment could not be confirmed. The chronic CT findings and clinical history were compatible with a remote orbitomaxillary injury, but the precise progression from the childhood injury to the adult deformity cannot be established.

The principal limitation of this report is the one-month clinical follow-up. The observed improvement in contour, equal Hertel measurements, and normal eyelid and ocular motility findings describe the early postoperative result. Longer follow-up is required to assess allograft incorporation, implant stability, sustained orbital symmetry, eyelid position and function, and patient-reported satisfaction.

## Conclusions

This case report describes combined orbital floor support, anterior maxillary reconstruction, and lower eyelid repair in an adult with a reported history of childhood dog-bite trauma. Reconstruction was associated with early improvement in periocular and midfacial contour, a reduction in the interocular Hertel difference from 4 mm to 0 mm, and a right CT-segmented orbital compartment volume decrease of approximately 1.0 mL. At one month postoperatively, ocular motility was full without diplopia, lower eyelid position and margin contour were normal, closure was complete, and epiphora was absent. These findings support an integrated anatomical assessment when planning delayed reconstruction. Longer follow-up is required to establish the durability of the result.

## References

[1] Chen HH, Neumeier AT, Davies BW, Durairaj VD (2013). Analysis of pediatric facial dog bites. Craniomaxillofac Trauma Reconstr.

[2] Foster MD, Hudson JW (2015). Contemporary update on the treatment of dog bite: injuries to the oral and maxillofacial region. J Oral Maxillofac Surg.

[3] Wei LA, Chen HH, Hink EM, Durairaj VD (2013). Pediatric facial fractures from dog bites. Ophthalmic Plast Reconstr Surg.

[4] Tu AH, Girotto JA, Singh N (2002). Facial fractures from dog bite injuries. Plast Reconstr Surg.

[5] Saadi R, Oberman BS, Lighthall JG (2018). Dog-bite-related craniofacial fractures among pediatric patients: a case series and review of literature. Craniomaxillofac Trauma Reconstr.

[6] Sandhaus H, Boakye EA, Johnson M (2023). Incidence of facial fractures in association with facial laceration from dog bites in the pediatric patient. Int J Pediatr Otorhinolaryngol.

[7] Selvi F, Stanbouly D, Stanbouly R (2022). Early childhood (0 to 5 years) presents the greatest risk for facial dog bites. J Oral Maxillofac Surg.

[8] Fourie L, Cartilidge D (1995). Fracture of the maxilla following dog bite to the face. Injury.

[9] Walker T, Modayil P, Cascarini L, Collyer J (2009). Dog bite-fracture of the mandible in a 9 month old infant: a case report. Cases J.

[10] Cottom H, Tuopar D, Ameerally P (2011). Mandibular fracture in a child resulting from a dog attack: a case report. Case Rep Dent.

[11] Baldi JR, Wolff de Freitas DA (2013). Mandible and zygomatic fracture in a 2-year-old patient due to dog bite. Craniomaxillofac Trauma Reconstr.

[12] Heitz C, Louzada GP, Conci RA, Rodrigues RL, Fritscher GG (2018). Primary repair of a complex panfacial fracture by dog bite. Plast Reconstr Surg Glob Open.

[13] Ellis E 3rd, Tan Y (2003). Assessment of internal orbital reconstructions for pure blowout fractures: cranial bone grafts versus titanium mesh. J Oral Maxillofac Surg.

[14] Dubois L, Steenen SA, Gooris PJ, Mourits MP, Becking AG (2015). Controversies in orbital reconstruction - I. Defect-driven orbital reconstruction: a systematic review. Int J Oral Maxillofac Surg.

[15] Avashia YJ, Sastry A, Fan KL, Mir HS, Thaller SR (2012). Materials used for reconstruction after orbital floor fracture. J Craniofac Surg.

